# P-1019. Incidence And Risk Factors for Invasive Fungal Infections in Pre-Transplant and Transplant-Ineligible Multiple Myeloma in the U.S.: A Claims Analysis (2017-2021)

**DOI:** 10.1093/ofid/ofae631.1209

**Published:** 2025-01-29

**Authors:** Daniel Rogers, Aubrey N Baker, Jianing Xu, Xianyan Chen, Andrés F Henao Martínez, Daniel B Chastain

**Affiliations:** Emory Healthcare, Atlanta, Georgia; University of Georgia College of Pharmacy, Albany, Georgia; University of Georgia, Athens, Georgia; UGA Franklin College of Arts and Sciences, Athens, Georgia; University of Colorado Anschutz Medical Campus, Aurora, Colorado; University of Georgia College of Pharmacy, Albany, Georgia

## Abstract

**Background:**

Current research on risk factors for invasive fungal infections (IFIs) in multiple myeloma (MM) has limitations due to heterogeneous patient populations, including post-transplant, or reliance on subgroup analyses. This study aimed to address this gap by evaluating the incidence of IFIs and identifying risk factors in patients with MM receiving treatment before or ineligible for transplant.

**Figure:**
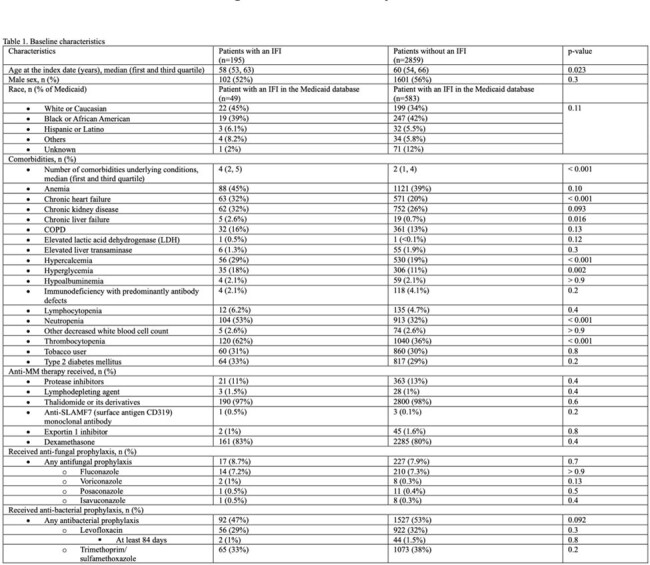

**Methods:**

We analyzed data from the Merative MarketScan Database (2017-2021) to identify adults (≥ 18 years) diagnosed with and treated for MM with proteasome inhibitors, lymphodepleting agents, thalidomide or derivatives, anti-SLAMF7 monoclonal antibodies, or exportin 1 inhibitors, with or without dexamethasone. We evaluated the incidence and risk factors for IFIs following anti-MM therapy initiation. All patients were followed for at least one year. Patients without an IFI were censored at the end of the study period.

**Figure:**
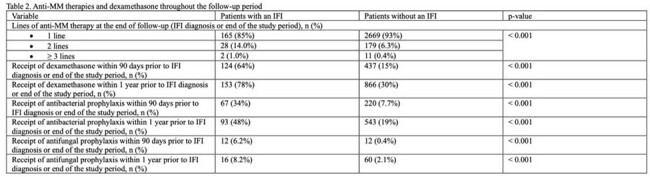

**Results:**

Among 3054 individuals with MM, 6% (n=195) were diagnosed with an IFI. Candidiasis was most common (87%), followed by pneumocystis (6.2%) and aspergillosis (3.6%). Patients with an IFI were younger with a higher burden of comorbidities compared to those without an IFI (table 1). Notably, neutropenia, thrombocytopenia, chronic heart failure, chronic liver failure, hypercalcemia, and hyperglycemia were significantly more common in the IFI group. Anti-MM therapies were similar between groups, with a high prevalence of both thalidomide or derivatives and dexamethasone (table 2). Antifungal prophylaxis was uncommon while nearly half of each group received antibacterial prophylaxis. Patients with an IFI were more likely to have received multiple lines of anti-MM therapy. Multivariate analysis identified recent dexamethasone use (HR 5.85, 95% CI: 4.08-8.40), neutropenia (HR 2.77, 95% CI: 1.87-4.11), and a greater number of anti-MM therapies within the preceding year (HR 2.15, 95% CI: 1.71-2.69) as significant risk factors for IFI.

**Conclusion:**

Candidiasis was the most common IFI in patients with MM. Younger age, higher comorbidity burden, and neutropenia were associated with IFIs. Additionally, recent dexamethasone use and a higher number of prior anti-MM therapies significantly increased the risk of IFIs.

**Disclosures:**

**All Authors**: No reported disclosures

